# Individual Differences in the Recognition of Facial Expressions: An Event-Related Potentials Study

**DOI:** 10.1371/journal.pone.0057325

**Published:** 2013-02-22

**Authors:** Yoshiyuki Tamamiya, Kazuo Hiraki

**Affiliations:** Graduate School of Arts and Sciences, the University of Tokyo, Tokyo, Japan; University of Bologna, Italy

## Abstract

Previous studies have shown that early posterior components of event-related potentials (ERPs) are modulated by facial expressions. The goal of the current study was to investigate individual differences in the recognition of facial expressions by examining the relationship between ERP components and the discrimination of facial expressions. Pictures of 3 facial expressions (angry, happy, and neutral) were presented to 36 young adults during ERP recording. Participants were asked to respond with a button press as soon as they recognized the expression depicted. A multiple regression analysis, where ERP components were set as predictor variables, assessed hits and reaction times in response to the facial expressions as dependent variables. The N170 amplitudes significantly predicted for accuracy of angry and happy expressions, and the N170 latencies were predictive for accuracy of neutral expressions. The P2 amplitudes significantly predicted reaction time. The P2 latencies significantly predicted reaction times only for neutral faces. These results suggest that individual differences in the recognition of facial expressions emerge from early components in visual processing.

## Introduction

The goal of the current study was to investigate individual differences in neural activity related to recognition of facial expressions. Some people are good at recognizing facial expressions, but others are not. Previous studies have suggested that not only individuals with brain damage [1] or schizophrenia [2] have difficulty in recognizing facial expressions but also that there are individual differences in recognition among healthy adults. For example, age [3], gender [4], personality, and mental states [5], affect recognition of facial expressions. However, the neural basis of these individual differences has not been clarified. The current study examined the role of individual differences in neural activity in the recognition of facial expressions.

Compared with other objects, face perception is unique and has been investigated extensively [6]. One functional magnetic resonance imaging (fMRI) study found that an area within the fusiform gyrus, implicated in face perception, responds more to faces than to other objects [7]. fMRI has been used extensively to examine differences in the localization of activation to face and nonface stimuli [8]. An integrative study using fMRI, electroencephalogram and magnetoencephalography showed similar activation to face stimuli [9]. Event-related potential (ERP) studies have investigated a signal that is sensitive to face processing, known as N170. N170 is a large, posterior negative deflection that follows the visual presentation of the picture of a face, peaking at right occipitotemporal sites at around 170 ms [10]. Previous studies have shown that N170 is modulated by various factors, including inversion [11], contrast [12], and emotional expressions [13], [14]. Past studies showed that N170 is also modulated by certain disease states [15]–[17]. For example, individuals with schizophrenia are impaired in their ability to accurately perceive facial expressions, and they display significantly smaller N170 amplitudes [18].

P2, which is the component that peaks at occipital sites at around 220 ms, is thought to reflect deeper processing of stimuli [19]. One previous study reported that compared to healthy adults, individuals with schizophrenia also had reduced P2 amplitudes [18].

There is also evidence that both N170 and P2 are modulated by expert object learning [19]. For example, an enhanced N170 was observed when bird and dog experts categorized objects in their domain of expertise relative to when they categorized objects outside their domain of expertise [20].

Given that N170 and P2 are modulated by expertise and learning, these components should be indices of individual differences in the recognition of facial expressions. Some studies reported correlations between behavioral measures and ERPs elicited by face stimuli [21]–[23]. However, the neural basis of individual differences to recognize facial expressions has not been clarified. The aim of the current study was to investigate individual differences in neural activity related to the recognition of facial expressions. We examined whether facial expression discrimination was associated with early ERP components: N170 and P2.

## Method

Ethics Statement: Written informed consent was obtained from each participant prior to the experiment. The ethics committee of The University of Tokyo approved this study.

Participants: Participants were recruited from local Japanese universities. Thirty-three healthy, right-handed paid volunteers (17 females and 16 males, aged 18–31 years, mean age = 22.3 years) participated in the experiment.

Stimuli: Stimuli consisted of photographs of facial expressions (2 females and 2 males posing angry, happy and neutral expressions) taken from the ATR Facial Expression Image Database (DB99). The database contains 4 females and 6 males and 3 pictures of each model posing each facial expression. The database also contains a result of a preliminary experiment, which is not published, to confirm validity of the database (see Supporting Information, Text S1). All stimuli were gray-scale pictures.

**Figure 1:**
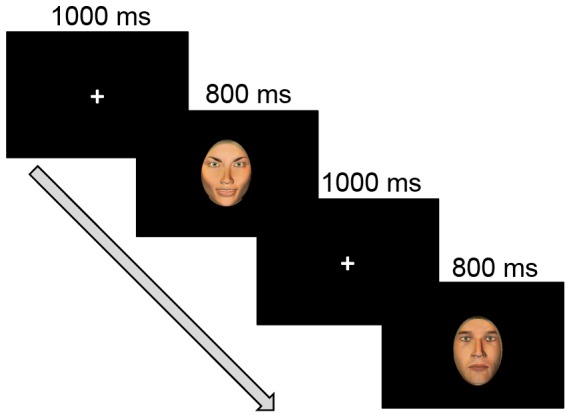
Illustration of the stimulus-presentation procedure used in the current experiment. Each trial started with a fixation followed by a facial expression. The images of faces shown here do not depict the actual stimuli but are intended only as examples.

Procedure: The protocol consisted of four blocks. Each block contained 120 facial stimuli, 40 photographs of each expression. The stimuli were presented in random order. All photographs were 5.5×8 cm. The mean luminance was equal across stimuli. Stimuli were presented on a black background on a 17″ CRT computer screen (EIZO FlexScan F520) at a viewing distance of 80 cm for 800 ms with an ISI of 1000 ms (see Fig. 1). Participants were told that they would be shown a series of photographs and were asked to respond to each facial expression by pressing certain keys. Before the actual experiment, participants practiced the task with a short training block that included stimulus examples not present during the experimental blocks. It took about 30 minutes to complete all tasks including short breaks.

**Figure 2:**
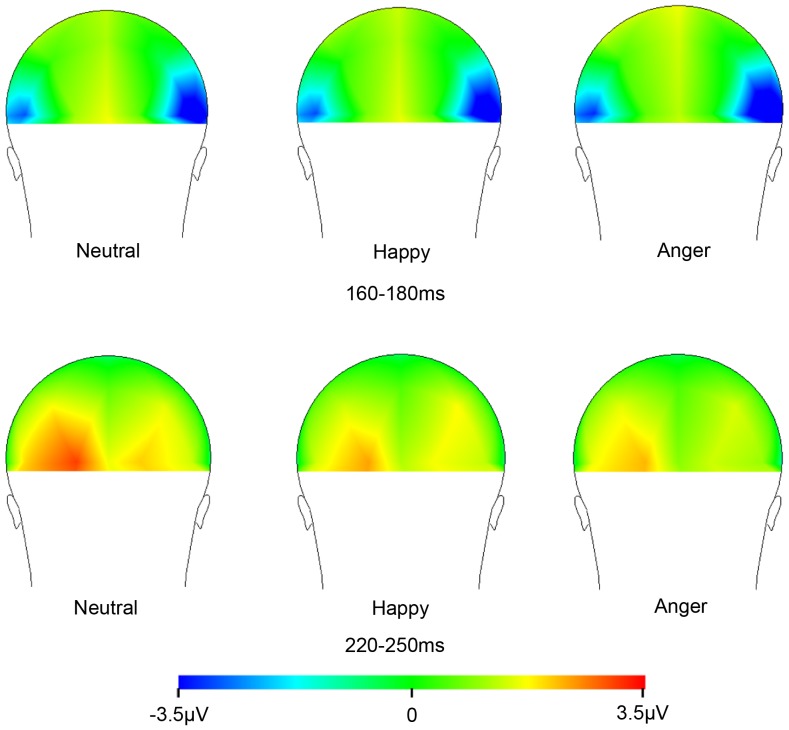
Topographical maps of N170 and P2 window for facial stimuli. Maps from a back view perspective. Negativity is shown in blue.

EEG recoding and data analysis: EEG was recorded from 65 electrodes with a Geodesic Sensor Net [24], sampled at 250 Hz with a 100 Hz low-pass filter. Electrode impedance was below 100 kΩ. The EEG recording system is high input impedance amplifier. The original study using the system [24] shows that with electrode impedance around 80 kΩ, the EEG data are still clear. Before recording, the experimenter severely checked the waveform on a screen. All recordings were initially referenced to the vertex and, later, digitally re-referenced to the average reference. In the off-line analysis, a 0.1–30 Hz band-pass filter was reapplied. All data were segmented into 800 ms epochs, including a 100 ms pre-stimulus baseline period, based on time markers for stimulus onset. All segments without eye movements and blinks and less than 75 microV in each channel were analyzed and baseline-corrected. Each component was measured at the area where the amplitude was maximal (see fig. 2). N170 was measured at electrodes P7 and P8 [10], [13]. P2 was measured at electrodes O1 and O2 [18], [19], [25]. Latencies were taken where the amplitude was maximal over each hemisphere, and amplitudes were measured at this latency. Peak amplitudes for each component were analyzed to compare the current results directly with relevant previous studies [15], [17], [19], [22]. A Greenhouse-Geisser adjustment of degrees of freedom, as well as a Bonferroni correction, was used when necessary.

Effect size was computed because it is recommended to include some index of effect size so that the reader fully understands the importance of the result [26], [27]

## Results

Behavioral: Trials which contained ±2 SD reaction time were excluded from the analyses. Participants showed greater accuracy in recognizing angry (85%) as compared to happy faces (80%: *F* (1.5, 52.8) = 4.9, *p* = .018, η_p_^2^ = .12; MS = 72.2, df = 49.8, *p*<.001). Other comparisons were not significant (neutral and angry faces, *p* = 1.0; happy and neutral faces, *p* = .062; respectively). No significant effects were observed regarding reaction time (angry = 586 ms, happy = 597 ms, and neutral = 592 ms; *F* (2, 68) = 2.3, *p* = .106, η_p_^2^ = .06; MS = 543.7, df = 68; neutral and angry faces, *p* = 1.0; happy and neutral faces, *p* = .344; happy and angry faces, *p* = .058; respectively).

**Figure 3:**
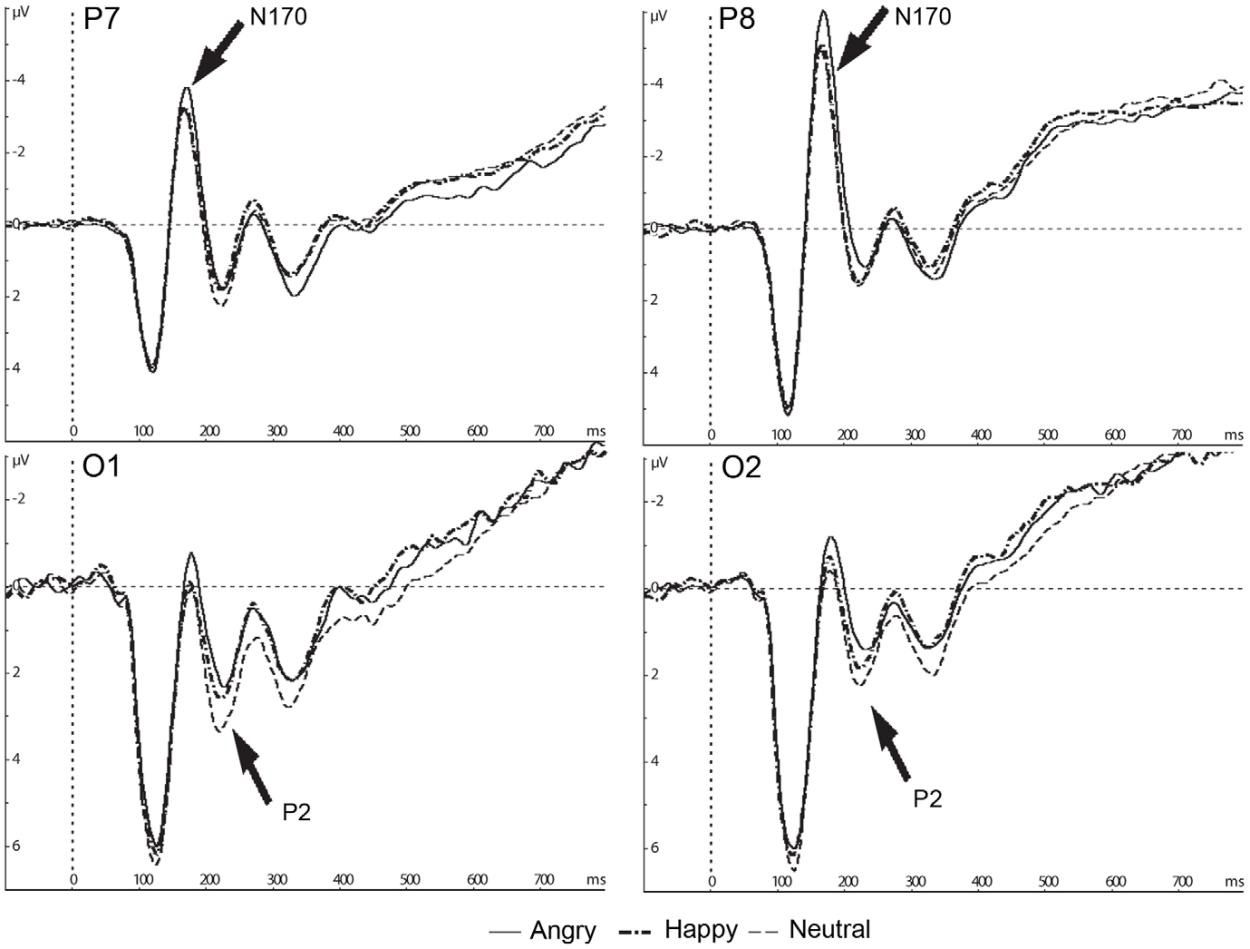
Grand average ERP waveforms of N170 and P2 amplitudes. Electrode site P7 and P8 for N170, and O1 and O2 for P2 are displayed. Negative amplitudes are plotted upward.

**Figure 4:**
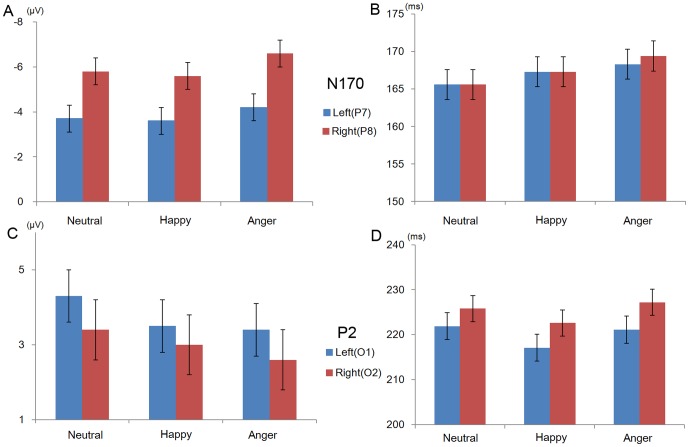
Mean and standard error of ERP components elicited by facial expressions. (A) Amplitudes of N170. Negative amplitudes are plotted upward. (B) Latencies of N170. (C) Amplitudes of P2. Positive amplitudes are plotted upward. (D) Latencies of P2.

ERP: Grand averaged waveforms and topography of ERPs are illustrated in Figure 2 and 3. Figure 4 and table 1 show means and standard errors of ERPs. The average number of noise free trials included in the grand average per participants was 407 trials (angry, happy and neutral expressions, respectively; 136, 135 and 136 trials). N170 amplitudes varied with emotion (*F* (1.6, 56.7) = 13.6, *p*<.001, η_p_^2^ = .28) and hemisphere (*F* (1, 35) = 13.5, *p*<.001, η_p_^2^ = .28). N170 amplitudes for angry faces were larger than those for happy and neutral faces (MS = 1.2, df = 56.68, *p*<.001; *p*<.001, respectively). A comparison between happy and neutral faces was not significant (*p* = .34). N170 amplitudes were also larger over the right hemisphere than over the left hemisphere.

**Table 1:** Mean and standard error of ERP components elicited by facial expressions.

	N170				P2			
	Amplitude(µV)		Latency(ms)		Amplitude(µV)		Latency(ms)	
	Left(P7)	Right(P8)	Left(P7)	Right(P8)	Left(O1)	Right(O2)	Left(O1)	Right(O2)
Neutral	−3.6(0.6)	−5.6(0.6)	165.6(2.1)	165.6(1.9)	4.3(0.7)	3.4(0.8)	221.9(2.8)	225.8(2.8)
Happy	−3.7(0.6)	−5.8(0.6)	167.2(2.2)	167.3(2.0)	3.5(0.7)	3.0(0.8)	217.1(3.0)	222.6(2.9)
Anger	−4.2(0.6)	−6.6(0.7)	168.3(1.6)	169.4(2.0)	3.4(0.8)	2.6(0.8)	221.1(3.0)	227.2(3.2)

An effect of emotion was seen on N170 latencies (*F* (2, 70) = 11.3, *p*<.001, η_p_^2^ = .24). N170 latencies for neutral faces were shorter than for angry faces (MS = 17.67, df = 70, *p*<.001). Other comparisons were not significant (happy and angry faces, *p* = .12; happy and neutral faces, *p* = .12; respectively).

For P2 amplitudes, there was an effect of emotion (*F* (2, 70) = 12.1, *p*<.001, η_p_^2^ = .26), with larger amplitudes elicited by neutral faces rather than happy and angry faces (MS = 1.02, df = 70, *p*<.001; *p*<.001, respectively). A comparison between happy and angry faces was not significant (*p* = .72).

P2 latency data also showed an effect of emotion (*F* (2, 70) = 3.5, *p* = .035, η_p_^2^ = .09); however, post-hoc analyses did not reveal significant differences between any two emotions (happy and angry faces, MS = 119.1, df = 70, *p* = .07; happy and neutral faces,, *p* = .177; angry and neutral faces, *p* = 1.0, respectively).

To examine the relationship between neural activity and recognition of facial expressions, stepwise multiple regression analyses were conducted. For each of six dependent measures (accuracy rate and reaction time for each facial expression), the amplitudes and latencies of ERPs were entered as predictors. This analysis allows unbiased, hypothesis-free comparisons of task-related activation patterns [28].

For two reasons, only N170 components over the right hemisphere were analyzed. First, consistent with previous studies [10], [12], N170 amplitudes were larger over right hemisphere sites than over left hemisphere sites. One fMRI study found greater activation in the right than left fusiform gyrus when viewing facial expression [7]. Past studies showed that face stimuli evoked a larger N170 over the right hemisphere [10], [12], [19], [23], [29]; but see some reports of no significant bilateral effect: [30]. Second, there was no clear difference in the coefficient of determination between a model that included both hemispheres and a model that only included the right hemisphere. In contrast to N170, there was no effect of hemisphere on P2 components, so we analyzed P2 over both hemispheres.

**Figure 5:**
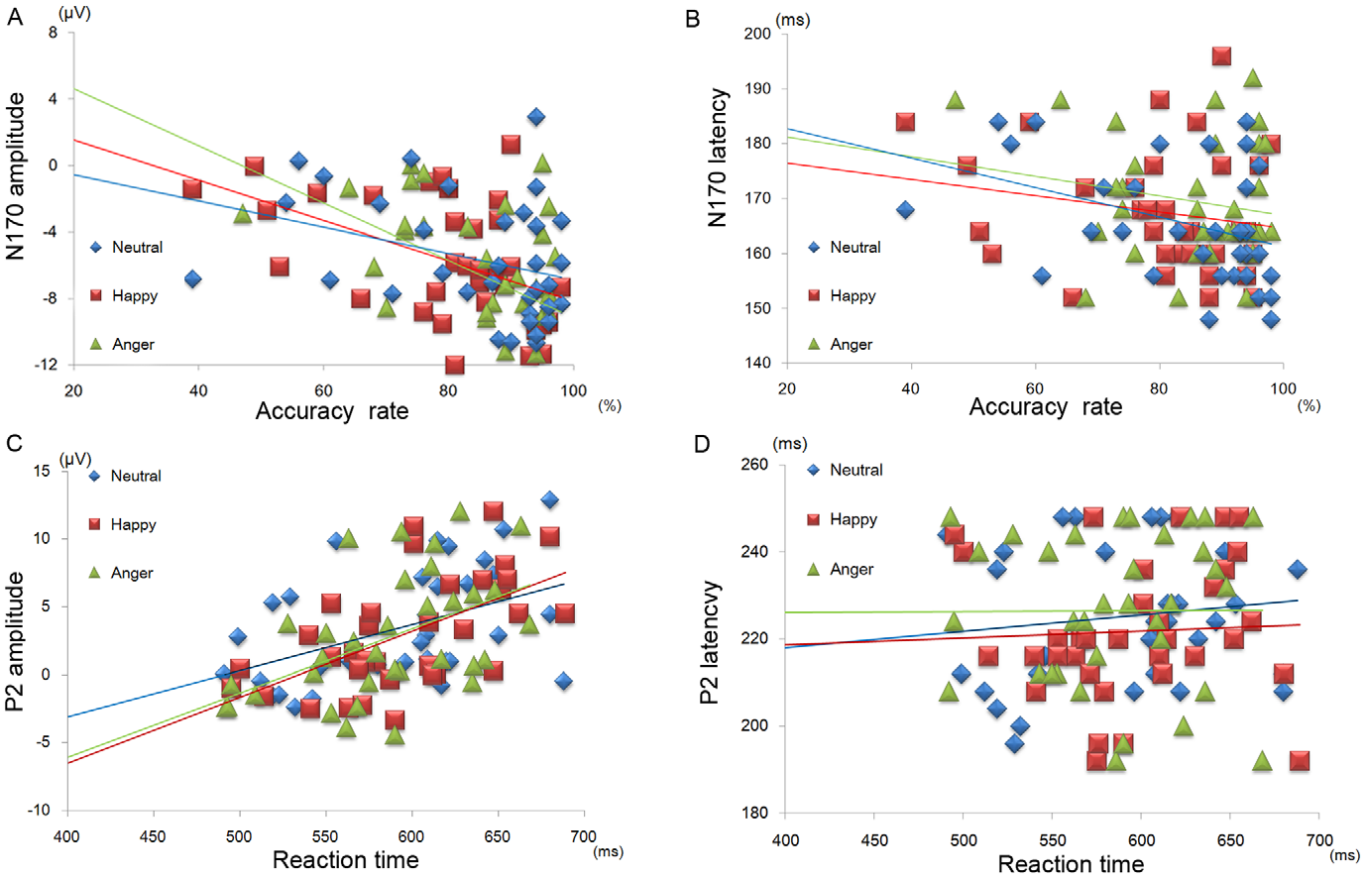
Scatter plot of the behavioral and ERP data. (A) Relationship between accuracy rate and N170 components: N170 amplitudes for angry and happy faces, and N170 latencies for neutral faces. (B) Relationship between reaction times and P2 components. Each plot indicates each participant, and each line depicts a regression line for a facial expression.

Figure 5 show the association between ERP components and the behavioral data. The N170 amplitudes were significant predictors for accuracy of angry and happy expressions, and N170 latencies were predictive for accuracy of neutral expressions (see Fig. 5a; angry, happy, and neutral, respectively; *β* = −1.41, 95% confidence interval (CI), −2.32 to −0.49, *R* ^2^ = .22, *p* = .004; *β* = −1.89, 95% CI, −3.14 to −0.63, *R* ^2^ = .22, *p* = .004; *β* = −.53, 95% CI, −0.99 to −0.08, *R* ^2^ = .14, *p* = .022; each *β is* unstandardized).

P2 amplitudes significantly predicted reaction times (see Fig. 5b; angry, happy and neutral expressions, respectively; *β* = 5.44, 95% CI, 2.27 to 8.61, *R* ^2^ = .26, *p* = .001; *β* = 6.71, 95% CI, 3.34 to 10.08, *R* ^2^ = .33, *p*<.001; *β* = 4.38, 95% CI, 0.26 to 8.5, *R* ^2^ = .33, *p* = .038; each *β is* unstandardized). P2 latencies significantly predicted reaction times only for neutral faces (*β* = 1.25, 95% CI, 0.17 to 2.33 *R* ^2^ = .26, *p* = .025; each *β is* unstandardized).

To detect violation of the data assumptions (multicollinearity among the predictors), pearson correlation coefficients were computed between the predictors in each model. The results showed no correlation higher than 0.70 and validated the models.

## Discussion

The current study investigated the relationship between individual differences in the recognition of facial expressions and neural activity among healthy adults. Results showed that the N170 and P2 components were related to recognition of facial expressions. N170 significantly predicted recognition accuracy; the larger N170 predicted higher accuracy rates. Some studies have suggested that N170 is a good neurophysiological index of face perception processes [10], [31]. Moreover, an enhanced N170 was found when bird and dog experts categorized objects in their domain of expertise [20]. Given these findings, the association between N170 and accuracy rates may reflect individual differences in the ability to discriminate facial expressions. Early emotional processing in young children differed from that observed in the adolescents, who approached adults [32]. In future studies, to examine whether the same pattern of individual differences are observed in children would be an interesting.

P2 was predictive of reaction times. Given that P2 had no direct link with accuracy rates, it is reasonable to assume that the P2 component did not directly reflect the categorization of facial expressions. A functional model of face cognition suggested the importance of distinguishing between speed and accuracy of face cognition [33]. P2 may reflect the activity related to speed of face cognition. As discussed above, P2 is also thought to reflect deeper processing of stimuli [19]. Therefore, our results indicate that variance in P2 is an index of individual differences in a deeper process such as second-order configural processing [22] and speed of face cognition that follows the categorization of facial expressions.

Many factors such as repetition [34]–[37] and familiarity [38] with facial stimuli used in a task affect the face processing. It is reasonable to assume that these factors had little effect on the current results. First, all stimuli were unfamiliar to participants. Second, the repetition effect diminished when different identities were presented continuously [39]. Facial stimuli used in the current study were taken from 4 different adults and presented in a random order. Moreover, all participants had the same task. Even if there was the repetition effect, all participants would be affected in the same way. Nevertheless, the present study showed individual difference in the recognition of facial expressions.

Our results are compatible with several clinical studies. Individuals with schizophrenia, who have great difficulty in recognizing facial expressions, have shown a specific reduction in N170 components and an increase in P2 components in response to faces, relative to healthy controls [18], [40]. The current study found that a low accuracy rate was linked to a reduction of N170 and that longer reaction times were associated with increases in P2 among healthy adults. These findings suggest that individual differences between clinical and non-clinical samples in the recognition of facial expressions may be continuous rather than discrete. To address this possibility, future studies should employ the same experimental procedure with clinical and non-clinical samples to examine potential discrepancies in facial processing and recognition.

Responses to neutral faces had a unique association with both N170 and P2. A previous study revealed significant differences between emotional faces and neutral faces only during an expression discrimination task. Results indicated that the difference was not due to the physical features of the stimuli but depended on cognitive aspects [25]. Difference between emotional and neutral faces may be elicited by different levels of arousal and not by the emotional content [14]. Our current findings may also reflect the unique cognitive processing of neutral faces.

Negative faces elicited larger amplitudes than positive faces. This result is consistent with the past studies [13], [14], though other studies reported that N170 amplitudes were larger following the positive as compared to the negative faces [16], [41]–[44]. The tasks and stimuli used in these previous studies are different from each other. Therefore, it is difficult to compare the effects directly and the result may be inconclusive. Because the focus of interest in the present study was on individual differences in the recognition of facial expressions, the present data do not deal explicitly with this question.

There are some limitations of the present study. One limitation relates to our stimuli. We employed three categories of emotional faces in order to avoid response conflicts among our participants. We are unsure whether a similar association would have been observed had we used other facial expressions. Since our interest was in recognition of facial expressions, all of the stimuli were faces. This raises the question as to whether our findings are specific to facial expressions or whether they can be generalized to other objects. This is unlikely to be the case, given that previous studies suggest a form of neural processing that is unique to face recognition in comparison to other objects [7], [10], [13,]. However, future studies should confirm the current findings with other types of stimuli in addition to faces.

Another limitation is the robustness of the current findings. Previous study suggested the importance to consider outliers in data [45]. In the current study, the effects of outliers would be small enough not to affect results. First, we excluded trials which contained ±2 SD reaction time in behavior data. Second, for EEG data, severe artifact rejection was conducted and more than 100 trials per condition were included in the grand average. However, the number of participants might be modest for a regression analysis. Future study will address these issues.

The aim of the current study was to investigate the relationship between individual differences in the recognition of facial expressions and neural activity. Therefore, we focused on the direct relationship between them and did not include any personal variable. Though the multiple regression models were significant, this does not mean that the models completely predict the recognition of facial expressions. Given that the cortical processing of facial emotional expression is modulated with many factors, such as executive function [46], social cognition skills [43] and disease [17], [18], [40], [41], further studies should build a more general model of recognition of facial expressions.

The present data sheds new light on individual differences in the neural activity associated with recognition of facial expressions. Models for face processing have been built [6], [47], [48] and previous studies investigated the neural basis [7] and time course [10], [13], [22]. Our findings suggest that individual differences in an important social skill, such as recognition of facial expressions, emerge during an early stage of information processing and give important implications for those models. Some people have difficulty recognizing facial expressions (e.g., individuals with brain damage [1], schizophrenia [18], neurodegenerative disease [49], [50], ADHD [41] and autism [51]). The current findings are an important first step toward providing potential explanations as to the mechanism(s) underlying facial emotion recognition impairments.

## Supporting Information

Text S1 **psychological evaluation of the ATR Facial Expression Image Database and the number of facial stimuli used in the current study.**
(DOC)
