# Supplementary material for: Individual Differences in the Recognition of Facial Expressions: An Event-Related Potentials Study
Source: PLoS One. 2013 Feb 22;8(2):e57325. doi: 10.1371/journal.pone.0057325 (PMC3579819; doi:10.1371/journal.pone.0057325)
Supplement: Text S1 — psychological evaluation of the ATR Facial Expression Image Database and the number of facial stimuli used in the current study. (DOC) [file pone.0057325.s001.doc]

Facial stimuli:

ATR, a developer of the ATR Facial Expression Image Database (DB99), conducted an experiment on psychological evaluation of the database. In the experiment, 27 university students evaluated expression intensities of each and classified them into seven categories – happiness, sadness, surprise, anger, disgust, fear and contempt – and into seven levels – from 1 (the least intense) to 7 (the most intense). The result clarified the intensities of each kind of facial expression.

The samples of photographs are shown on the ATR web page (http://www.atr-p.com/face-db.html).

The database has been used in previous studies [1 - 3] and no study reports invalidity of the database.

Based on the result of the experiment conducted by ATR, we chose F03-f00-e00-AO-2, F16-f00-e00-AO-1, M02-f00-e00-AO-1 and M05-f00-e00-AO-2 for angry faces (intensity rating for angry respectively, 6.519, 5.815, 6.444 and 6.333), F03-f00-e00-AO-2, F16-f00-e00-SO-2, M02-f00-e00-SO-3 and M05-f00-e00-SO-3 for happy faces (intensity rating for happy respectively, 6.296, 6.407, 6.444 and 6.333) and F03-f00-e00-NE-1, F16-f00-e00-NE-1, M02-f00-e00-NE-1 and M05-f00-e00-NE-1 for neutral faces (each intensity rating for each expression is under 3.4) from the database.

References

1. Kamachi M, Bruce V, Mukaida S, Gyoba J, Yoshikawa S, et al. (2001) Dynamic properties influence the perception of facial expressions. Perception 30: 875–887.
2. Nishitani S, Doi H, Koyama A, Shinohara K (2011) Differential prefrontal response to infant facial emotions in mothers compared with non-mothers. Neuroscience research 70: 183–188.
3. Suzuki M, Noguchi Y (2012) Reversal of the face-inversion effect in N170 under unconscious visual processing. Neuropsychologia 51: 400–409.
